# BJPsych Bulletin In Memoriam List 2023

**DOI:** 10.1192/bjb.2022.82

**Published:** 2023-02

Atkinson, Rosemary Wyn, Fellow ~ Over 40 Years member

Beaumont, George, Fellow ~ Over 40 Years member

Beynon, Peter John, Fellow ~ Over 40 Years member

Bewley, Thomas Henry, Honorary Fellow Previously Member

Bilal, Ali Mustafa, Member ~ Over 40 Years member

Birtchnell, John Anthony, Fellow ~ Over 40 Years member

Black, Ann Dora, Fellow

Bolton, Anne, Fellow ~ Over 40 Years member

Bosanquet, Camill, Fellow

Brafman, Abrahao Henrique, Member ~ Over 40 Years member

Burnfield, Alexander John, Fellow, Over 40 Years member

Butler, Pieter Ormonde G., Member ~ Over 40 Years member

Candy, Julian, Fellow ~ Over 40 Years member

Carter, David Martyn, Fellow

Chen, Leslie Ting Yeung, Member ~ Over 40 Years member

Clavering, Emily Frances, Member

Cohen, Philip Henry, Member ~ Over 40 Years member

Crawford, Robert John Mackay, Affiliate ~ Over 40 Years member

De-Albuquerque, Afonso, Affiliate ~ Over 40 Years member

Diamond, John, Member ~ Over 40 Years member

Dick, Donald Hugh, Fellow ~ Over 40 Years member

Douglas, Neil, Honorary Fellow

Eastman, Nigel Lyons Gwynne, Fellow

Fraser, William Irvine, Fellow ~ Over 40 Years member

Gimbrett, Roy, Member

Halim, Mohammed Abdul, Fellow ~ Over 40 Years member

Hinchliffe, Mary Katherine, Fellow ~ Over 40 Years member

Idogun, Onakome Ometejohwo, Affiliate

Jouhargy, Wafaa, Member

Jones, Dewi, Fellow ~ Over 40 Years member

Jungalwalla, Hoshang Nowshire K., Member

Kamala Chandrasekhar, Turuvekere Veerappa Kamala, Fellow ~ Over 40 Years member

Kenrick, Jeremy Martin Tyrrell, Member ~ Over 40 Years member

King, Rosalind Ann, Member ~ Over 40 Years member

Launer, Michael Andrew, Member ~ Over 40 Years member

Lee, Alan Stuart, Fellow ~ Over 40 Years member

Lester, Marc Timothy, Member

Lind, Berthold, Affiliate

MacKinnon, Ranald Scott, Fellow ~ Over 40 Years member

Malomo, Idowu Oladunjoye, Member ~ Over 40 Years member

Millar, John, Member ~ Over 40 Years member

Miller, Dennis Harold, Fellow ~ Over 40 Years member

Morgan, Janice, Member

Mukhopadhaya, Kaushik, Member

Mullan, Nuala Martina, Member

Pearce, Stephen, Member

Ravey, Moira, Fellow ~ Over 40 Years member

Riahinejad, M Reza, Fellow ~ Over 40 Years member

Richmond, Peter William, Member ~ Over 40 Years member

Rutter, Michael Llewellyn, Fellow

Sethi, Padam Chand, Member ~ Over 40 Years member

Shah, Dhanji Damji, Affiliate

Sharma, Kishan Lokesh, Member

Sheldon, Alan Peter, Member

Sinclair, Allan, Fellow

Slattery, Zoe Taschereau, Fellow ~ Over 40 Years member

Soundararajan, Portonovo Chockalingam, Member ~ Over 40 Years member

Spelman, Michael Stanway, Fellow ~ Over 40 Years member

Sternberg, Michael Paul, Member ~ Over 40 Years member

Stirland, John David, Fellow ~ Over 40 Years member

Todd, Norman Alexander, Fellow

West, Donald James, Fellow

White, Denis Michael Drummond, Fellow ~ Over 40 Years member

Wood, Robert Anderson, Honorary Fellow

Worrall, Neil Bevan, Fellow ~ Over 40 Years member

